# A Critical Analysis of Intimate Partner Sexual Violence in Iran

**DOI:** 10.3389/fpsyg.2019.02729

**Published:** 2019-12-06

**Authors:** Azam Naghavi, Shoale Amani, Marzieh Bagheri, Jan De Mol

**Affiliations:** ^1^Department of Counseling, Faculty of Education and Psychology, University of Isfahan, Isfahan, Iran; ^2^Department of Psychology, University of Mohaghegh Ardabili, Ardabil, Iran; ^3^Psychological Sciences Research Institute, Université catholique de Louvain, Louvain-la-Neuve, Belgium

**Keywords:** intimate partner violence, sexual violence, empowerment, women, Iran

## Abstract

According to global data, intimate partner violence and its corresponding impact threaten the lives of almost 35% of women at some point in their life. The aim of this research was to explore the effects of intimate partner sexual violence on women’s sense of self-efficacy when it comes to speaking out against violence and seeking help. In-depth interviews and a thematic analysis approach were employed to collect and analyze the data. The participants were 10 women with experiences of intimate partner sexual violence. They were selected through purposive and snowball sampling. Two main themes were drawn from the data, including Exposure and Empowerment. Exposure refers to the type of violence women have experienced and its physical and emotional effects; and Empowerment refers to factors women considered as giving them the courage to speak out against perpetrators, to seek help from others, or to refrain from doing either. It is concluded that Iranian women are not passive when exposed to intimate partner sexual violence, and social support, mainly from family and friends, was a pathway to feelings of empowerment; without this support, women’s emotional health is put in jeopardy. Due to the importance of social networks in creating a sense of empowerment, it is recommended that professionals involved in cases of intimate partner sexual violence create an alliance with the women’s families and friend and educate them on how to prevent violence or offer help before the violence takes its toll on woman’s emotional and physical wellbeing.

When my first child was 7, I got so tired of him [my husband] and I refused to have sex with him anymore. One time, I woke up in the middle of the night and saw that he was watching porn and masturbating. When he saw I was awake, he became furious and threatened me with a knife, threatening to do whatever he wanted otherwise he would kill me and my daughters. He said, “Do this or I will rape our daughter.” I had to do that [what they saw on porn clip] … I didn’t have any choice… It was hard…

Intimate partner violence (IPV) is a global public health issue and has been found to result in disability, suicide, or homicide for many women across the globe. According to the World Health Organization (2013), almost 35% of women around the world have faced physical and/or sexual abuse at some point in their lives. In contrast to the common misnomer, the majority of violence against women happens in the context of an intimate relationship. Intimate partner sexual violence is one form of violence against women that is often overlooked, while according to the UN’s facts and figures (United Nation, 2017) at least one in ten women in the world have experienced forced intercourse or other forced sexual activities at the hands of a partner at some point in their lives. Intimate partner sexual violence is defined as “being physically forced to have sexual intercourse when you did not want to, having sexual intercourse because you were afraid of what your partner might do, and/or being forced to do something sexual that you found humiliating or degrading” (World Health Organization, 2013, p. 13).

Intimate partner sexual violence (IPSV) is grave in nature, and it can cause several mental and/or physical health problems or disabilities. Researchers have found that women who have experienced sexual violence by a partner might suffer from PTSD (Bennice et al., 2003; Kessler et al., 2017; Zakrison et al., 2017; Honda et al., 2018), depression (Davhana-Maselesele et al., 2014; Honda et al., 2018), anxiety (Jaquier et al., 2015; Honda et al., 2018), substance and alcohol abuse (Decker et al., 2014; Jaquier et al., 2015; Hahm et al., 2017; Pengpid et al., 2018), somatic symptoms (Honda et al., 2018), sleep problems (Issahaku, 2015), and suicidal ideation and/or attempt (Sedziafa et al., 2016; Honda et al., 2018; Pengpid et al., 2018). They might also experience an unwanted pregnancy, abortion, being infected with HIV, and experience other forms of physical issues such as facial injuries, physical weakness, hypertension, and genital problems (Sedziafa et al., 2016).

There are several theories that explain the causes of violence against women. Larsen (2016) summarized these theories into three broad categories, including psychological explanations of IPV such as social learning theory, theories that focus on the biological issue such as neurochemical mechanisms of violent behaviors, as well as criminologists’ and sociological theories such as the feminist approach and family conflict theories. Larsen argues that IPV is a multifaceted phenomenon that needs to be researched taking into consideration its individual-level factors, such as women’s resources, and societal-level factors, such as patriarchal structure and social policy.

In “The roots of intimate partner violence,” Chester and Dewall (2018) argue that interpersonal factors, including dehumanization of women, social rejection, and infidelity, as well as intrapersonal factors such as psychopathology of perpetrators, lack of self-control, and substance abuse are among the reasons why violence against women is committed. Cultural and social justifications of violent acts are also considered important risk factors in IPV. In researching forced sex within marriage in India, Sinha (2017) found that 19% of participants justified wife-beating if the female partner refused to have sex. This incident is more likely to happen in rural areas, and among men with low socioeconomic status, illiterate people, men in an unhappy marriage, marriages with poor communication, and men who embrace an authoritarian role and see wife-beating as normal. Jeffrey and Barata (2017) found that women might also justify sexual violence on the basis of male sexual desires.

According to the United Nation (2017), despite the potentially serious health problems associated with IPV, less than four in ten women who have experienced violence seek help, and this number drops down even lower in IPSV cases. Gutzmer et al. (2016) found that some of their African-American participants never talked about their experiences of sexual coercion with others. Cultural and societal scripts often predict who will seek help and from whom. Believing in the concept that a woman should be ready to have sex whenever her partner asks for it contributes to accepting sexual violence and not reporting its incidence to the authorities (Lynch et al., 2017). In some situations, asking for help does not resolve the issue. Adinkrah (2017) analyzed 25 cases of homicide and attempted homicide in Ghana where events were triggered by a wife’s refusal of sexual intercourse and found that some pastors often suggested that women should go back to their home and not refuse to have sex with their husband in order to prevent marriage breakdown. In fact, women in Ghana are socialized to respond to their husbands’ sexual needs. Women’s perceptions of communities’ tolerance of sexual violence have been shown to be an influential factor in the help-seeking behavior of refugee women in Uganda (Odwe et al., 2018).

Disclosing IPSV is highly culturally bonded and in many countries is highly stigmatized. To ensure the fifth goal of the UN’s global goals in the next 15 years, which is to “eliminate all forms of violence against all women and girls in the public and private spheres, including trafficking, sexual and other types of exploitations,” it is important to learn more about help-seeking behaviors of women who have experienced IPSV in different cultures. According to the World Health Organization (2013), 37% of women who have ever had a partner in Eastern Mediterranean countries, including Iran, have experienced physical and/or sexual violence; that is the second-highest prevalence of IPV after the South-East Asian region.

## Cultural Practices Around Marriage and Family in Iran

Every culture has its own basic categories and patterns to organize ideas and values. Explaining Iranian culture is beyond the scope of this paper, but here we briefly look at the relevant cultural patterns around marriage and family life in Iran.

Iran, with an area of 1.6 million sq. km, is located in the Middle East and has borders with Iraq and Turkey to the West; Afghanistan and Pakistan to the East; Azerbaijan, Turkmenistan, and Armenia to the North; and with the Persian Gulf and Oman Sea to the South. According to the latest census in 2011, the population is 75 million people, of whom 49.6% are women (Statistical Center of Iran, 2012). Several ethnic groups including Persians, Arabs, Kurds, Turks, Lur, Azeri, Balouchi, Turkmen, and Gilaki are living in Iran. Persian (Farsi) is the official language and Islam is the religion of almost 98% of Iranians. There are also small populations of Zoroastrians, Christians, Jews, and Baha’i residing in different parts of Iran. The majority of Iranians (71%) live in urban areas (US Department of State, 2011).

With such a diverse range of ethnic groups and diverse religious practices, it is not surprising that each region of Iran has its own rituals and customs around marriage and family life, but heterosexual marriage is the only acceptable and legal form of marriage in Iran. Generally, in Muslim families, the man proposes to the woman and must seek the permission of the bride’s parent, if she is a virgin (Price, 2009). Marriage is considered to be a registered contract between two parties with different rights and responsibilities. According to this contract, the husband should support his wife and pay her *mahrye* upon her request and the wife should obey her husband. Under the Islamic practice, the husband has also the right to polygamy, the right to custody of the children, and the right to divorce.

All marriages should be registered in Iran, and the husband and wife should sign a marriage deed that includes the amount and type of *mahrye* (as present) that a husband should pay to his wife upon her request and the conditions that give the women the right to divorce, such as serious drug addiction of the husband, his imprisonment for over 5 years, and insanity. There is one condition called the “other condition,” which women can use as an opportunity to maintain their right to work, to study, to choose the place they live or even to divorce; otherwise, the husband can prohibit all of these. Marriage in Iran is not a union between just two people; the new bride and groom place themselves in a network of kin and are expected to maintain ties with their extended families (Bastani, 2007).

Iran traditionally has been a patriarchal society. In patriarchal societies, boys and girls are prepared to practice traditional gender roles from early childhood (Ghazizadeh et al., 2018). This can be done through formal and informal education. Gender socialization teaches boys to be the head of the family, to be active, aggressive, competitive, and independent, and teaches girls to be kind, peacekeeping, and obedient (Danesh et al., 2016). This is the process that Simone De Beauvoir considered as the factor that allows for continuing male domination over women (De Beauvoir, 1953). According to Foroutan’s analysis of the Iranian primary school textbooks (Foroutan, 2010a,b), these books are gender-biased, showing predominantly males, male sports, male works, and male photographs. Men in these books are depicted as the head of the family and the main breadwinner while women are generally shown as mothers who are taking care of their children or doing housework.

In patriarchal societies, men’s violence is normalized and women learn that violence is part of everybody’s private lives (Ghazizadeh et al., 2018). Therefore, they are expected to keep silent and be obedient and committed to their marital life. When women protest against domestic violence, they are excluded from society, often even by their female counterparts (Danesh et al., 2016). In such situations, some women perceive that they have no agency or power to change their husband’s abusive behaviors (Mohammadkhani et al., 2010).

Instilling gender roles seems to be very successful in some parts of Iran, for example in a study in one small town in Iran, Mohammadi and Mirzaei (2011) found that 67% of men and 70% of women agreed with stereotyped gender roles. In such a society, it is not surprising that violence has become normalized, and women do not speak against violence for fear of what other people might say behind their backs, fear of losing their children, lack of support from their family, and lack of knowledge of or access to support services (Tavasoli and Monirifar, 2009).

Guilt and embarrassment are two powerful tools that prevent women from disclosing violence in the family, especially when women feel that social support would not be available (Kabiri et al., 2018). Women are expected to preserve their family’s honor by keeping their problems within the family and they are often advised to tolerate any difficulties in their marriage and resolve problems by themselves. In the case of violence, it is not generally accepted to call the police, and women are expected to either keep silent or to seek help from elders in their community, or alternatively from counselors or psychologists. Several Iranian studies have shown that a lack of social support is strongly related to increased domestic violence (Babaeifard and Heydarian, 2014; Nikokar et al., 2014; Heidarinejad and Navah, 2018; Kabiri et al., 2018).

Contemporary Iranian society is experiencing a complicated time. On the one hand, patriarchal ideas, norms, and rules still fight to stay alive; on the other hand, through modernization and urbanization in recent decades, the structure of the Iranian family has gone through some radical changes including transformation of the extended family into a nuclear one and a movement from arranged marriages to non-arranged, an increase in the mean age of marriage from 19.8 years in 1986 to 23.2 years in 2006 (Saadat et al., 2010), an increase in the rate of uptake in women’s education and employment (Mohajerani, 2010) and a reduction in the fertility rate from 5.6 in 1978 to 1.7 in 2010 (United Nations Children’s Fund, 2010; Kohan et al., 2012).

Iranian women now have entered the formal social, political, and economic world and request equal rights (Yazdekhasti and Shiri, 2008; Nikpay and Pooya, 2012). The traditional discourse is no longer the dominant one and with wider access to social media, the influence of global values such as human rights and the growing women’s movement, Iranian women started to have a voice against violence. Creating NGOs to support women, improving family laws, opening counseling centers are some of the movements that could not have been successful without women’s support (Yazdekhasti and Shiri, 2008).

It is worth mentioning that despite all the prescribed cultural roles for women, Iranian women are neither passive nor oppressed by gender stereotypes or cultural rules. In contrast, since the Iranian revolution in 1979, women have gained more agency and power in decision-making (Mahmoudian, 2005) through more education and employment. The number of female admissions at an undergraduate level has increased from 31% in 1970 to around 60% since 2000 (Rezai-Rashti, 2011) and the rate of female employment has increased from 9.1% in 1996 to 14.9% in 2016 (Modaresialam et al., 2017).

## Intimate Partner Violence in Iran

According to Iranian studies, there is not an officially recognized prevalence of IPV in Iran and existing reports are from cross-sectional or meta-analytical studies. For example, in a study about prevalence of IPV among married women in the southeast of Iran, Ansari et al. (2012) found that 5.4% of women had experienced physical abuse, 20% had experienced emotional abuse, and around 10% had experienced sexual abuse. The prevalence is much higher in a paper published by Fallah et al. (2015), with reports of 32, 49, and 33% for physical, emotional, and sexual violence, respectively, at the hands of an intimate partner among 237 women in the northern region of Iran.

There are two meta-analyses of Iranian papers that have found a prevalence close to the World Health Organization’s, 2013 report. Bagrezaei et al. (2017), after an analysis of 25 papers with over 9,000 participants from all parts of Iran, found 37, 52, and 11% of Iranian women have experienced intimate partner physical, emotional, and sexual violence, respectively; and Haj Nasiri et al. (2017) showed that among 27,185 participants in 52 papers, 45, 59, and 32% have experienced intimate partner physical, emotional, and sexual violence, respectively. These studies show that IPV happens in all ethnic groups in Iran and there is not a significant relationship between IPV experiences and ethnicity.

Various studies have revealed significant relationships between the following factors and IPV in Iran: a husband’s employment and socioeconomic status, the number of children in the household, a family’s housing status (Ansari et al., 2012; Ahmadi et al., 2014), a lower level of emotional quotient (EQ) in a husband (Jafarian et al., 2016), being a housewife, experiencing violence, levels of social support, family interference, social capital of the family (Bagrezaei et al., 2017), and cultural norms in the socialization process, such as accepting patriarchal views (Danesh et al., 2016; Kianfard et al., 2017; Sohrabzadeh and Mansourian, 2017).

Iranian studies have also shown that women who have experienced any form of IPV might suffer from mental health problems such as depression (Dasarband et al., 2017) and PTSD (Rashti and Golshokouh, 2010) that needed further treatment and psychological rehabilitation. Feelings of low self-worth (Shoakazemi, 2016) and dehumanization (Shahbazi et al., 2018), psychosomatic complaints, sexual problems, increased tendency to use/abuse drugs and alcohol, divorce, and infidelity (Noori et al., 2016) were some other consequences of IPV uncovered in Iranian studies.

Although there is a high prevalence of IPV in Iran, with a considerable amount of resulting physical and mental health problems, there is a dearth of knowledge about experiences of IPSV and help-seeking behavior of Iranian women. One exception is Kianfard et al. (2017), who performed a needs assessment around domestic violence against women in Ahvaz. In this study, researchers asked why some women tolerate IPV and found that participants replied with answers such as low economic support, lack of awareness about women’s rights, and low perceived self-efficacy as reasons for tolerating violence. But this study also did not focus on IPSV; nor did it focus on help-seeking behavior as one of its main points. Another relevant study about IPSV is Rezaei and Abdar (2018), who performed qualitative research on sexual violence within families. This study focused on the types of sexual violence and their consequences but did not give any explanation about help-seeking behavior. To respond to the global call to eliminate all forms of violence against women, and in an attempt to fill the gap in cultural data about both IPSV in Iran and help-seeking behaviors of Iranian women, this paper will explore the following questions: How do Iranian women experience IPSV? How does IPSV affect Iranian women’s sense of self-efficacy when it comes to speaking out against violence and seeking help? And which factors would help them to seek help?

## Materials and Methods

Thematic analysis (Braun and Clarke, 2006) has been used to analyze in-depth qualitative interviews with participants who have experienced IPSV. Thematic analysis has been widely used in psychology, health and social sciences since the ground-breaking paper by Braun and Clarke (2006) (Clarke and Braun, 2015). They argue that thematic analysis is a method that can be used for identifying and analyzing patterns in qualitative data (Braun and Clarke, 2013), and, since it does not rely on any specific methods of data collection or theoretical standpoint, thematic analysis can be used to analyze almost any kind of data, and to answer almost any research question (Braun and Clarke, 2013).

### Participants

There is a growing body of literature on sampling methods for “hard-to-reach” and hidden populations, i.e., groups of people that are difficult to sample because of their physical or geographical locations, or their unwillingness to participate in research for legal or ethical reasons. These groups of people tend not to aggregate into lists of inhabitants who can be targeted for sampling (Goodman, 2011). To access this kind of population, researchers employ purposive and opportunistic sampling (Liamputtong, 2009; Hennink et al., 2011), methods that have been used in this study as well.

The first and most important inclusion criterion was the experience of IPSV. This is considered a sensitive issue in many settings, and therefore survivors seem to make up a hard-to-reach population. Ten women who were currently experiencing or had previously experienced IPSV in their current or past marriage were selected through purposive and snowball sampling. These women were recruited from two counseling centers, as well as a healthcare center. According to the World Health Organization (2013), in many countries, sexual violence is highly stigmatized and, although it is among the most severe forms of violence, many women suffer in silence. Iran is not an exception. As can be seen in Table 1, the majority of the participating women (seven) came from arranged marriages, while the other three marriages were love matches. The participants were between 26 and 45 years old and six of them were 18 or younger at the time of marriage. Four participants had been divorced for between 2 and 9 years, four of them had applied for a divorce, and two were living with their husband at the time of the interview. Only two participants’ husbands had had a higher education, five had primary or secondary school education, and three were illiterate; four husbands had addictions to drugs or alcohol.

**Table 1:** Participant’s demographic data.

	Pseudonym	Age	Age at marriage	Marital status	Number of children	Type of marriage
1	Mina	25–30	16–18	Divorced	0	Arranged
2	Sara	30–35	16–18	Divorced	2	Arranged
3	Sonia	25–30	16–18	Divorced	0	Love
4	Tara	30–35	25–30	Divorced	1	Love
5	Tina	25–30	16–18	Applied for divorce	2	Arranged
6	Sima	40–45	16–18	Applied for divorce	2	Arranged
7	Tania	25–30	20–25	Applied for divorce	0	Love
8	Tala	40–45	16–18	Applied for divorce	3	Arranged
9	Kiana	30–35	20–25	Married	0	Arranged
10	Soha	40–45	19–20	Married	2	Arranged

### Procedures

The University of Isfahan Ethics Committee for the Department of Counseling granted ethical approval and participants received a face-to-face explanation about the research aims and objectives, issues of confidentiality, informed consent, and dealing with possible adverse outcomes of the interview. Although not recording the sessions made the work more difficult, at the request of some participants, four interviews were not recorded, with the interviewers instead taking notes during the session. To ensure confidentiality, pseudonyms have been used and other identifiers such as the occupations of the women and their husbands have been removed.

Participants were selected from counseling and healthcare centers through purposive and snowball sampling. In-depth interviews were conducted with participants at a time and location of their choice. AN conducted two interviews and supervised the other interviews, which were done by the SA and MB. The interviews took 1–2 hours, but there were also telephone conversations that were subsequently conducted in order to make clarifications. Four participants did not consent to their voices being recorded but all agreed that the interviewers could take notes during the interview. To select the best questions for the purpose of the study, we have talked with the experts in the field and also reviewed the literature with this regard. General questions that could answer the main aims of the research were selected and an interview guide was built. All interviewers used the interview guideline, which consisted of four main questions: “Could you please speak about your relationship with your partner?,” “Could you please tell me what kind of violence you experienced?”, “Have you ever talked with anybody or sought help?”, and “From whom and when?”

### Data Analysis

Braun and Clarke’s thematic analysis approach (Braun and Clarke, 2006, Clarke and Braun, 2015) was employed for analysis of the data. This method is perceived to be a key analyzing method in qualitative research (Braun and Clarke, 2006). In an iterative way, data were analyzed based on the Braun and Clarke’s six-step method. There were discussions about codes and themes between the authors. The last author supervised the coding process and analysis of the data. There were two important themes: Exposure and Empowerment.

Exposure describes the different kinds of IPSV that women had experienced, and empowerment is about factors the women perceived as giving them the courage to speak out against perpetrators and to seek help from others, or to refrain from doing either. Based on my [the first author’s] experiences as an Iranian woman, as well as a therapist and qualitative researcher working with women in domestic violence cases, these results seem important. In Iranian culture, as in many other cultures around the world, marriage is an important stage of life and having a successful marriage that lasts until death is a wish many parents have for their children. On the other hand, as discussed above, keeping everything within the family and solving issues, as a family is a norm and women, in particular, are supposed to keep the family’s honor and to not disclose problems to outsiders. This norm will become even more complicated when there is a sexual problem involved, because, as in many places, speaking about sexual issues is a taboo in Iranian culture.

The information that women in this study disclosed about the types of sexual violence they had experienced is relatively unique as there is only one piece of Iranian qualitative research that has explored what kind of marital sexual violence women might experience in Iran. However, because of the qualitative nature of the study, we cannot assume these types of sexual violence are common to all Iranian women, nor that we cover all types of violence women might experience. Another theme of the research might be interesting as women, especially Muslims, are sometimes considered as oppressed figures, who need to be saved by others. However, in our observations, a lot of women in Iran have the capacity to and capability of helping themselves if they receive enough social support. Many women under pressure choose to speak out against their perpetrators if they know that there is a shelter out there for them. One problem is the cultural scripts that prevent women from speaking out and letting other people know about the violence they are experiencing or the trouble they must go through to make others believe that the violence is serious. It takes some women several years to finally prove the gravity of their situation, but when their close social networks believe it, they often rush to help.

## Results

How participants experienced IPSV and how they sought help are contextualized based on cultural practices around marriage and divorce in Iran. There is a famous Iranian expression that says: *a girl goes to her husband’s house wearing a white bride’s dress and comes out wearing a white shroud*, meaning that you should live with your husband forever, irrespective of any difficulties and conflict you might experience. Although the younger generation often does not follow such cultural scripts completely, they might face sanctions from the older generation in order to encourage them to practice the rules and customs. Therefore, when women face marital conflict, they might be asked to maintain their family’s honor and stay with their husband, even if the conflict turns to violence. Two important themes were uncovered in the interviews: exposure and empowerment. All of the participants talked not only about the different types of sexual violence they had experienced and the effects of it (exposure), but also about a variety of methods they had adopted to save themselves from violent situations or about factors that had hindered help-seeking (empowerment).

### Theme 1: Exposure, Types of Sexual Violence, and Its Effects

#### Sub-theme: Forced Sex

The majority of participants had experienced an arranged marriage at age 17 or 18, and experiences of forced intercourse or other unwanted sexual activities were common among all participants. Mina, who was 26 years old at the time of the interview, got married when she was 18. She was in love with someone else, which displeased her parents. After a long period of conflict with her parents, she agreed to get married to someone she did not love. During the engagement period, she asked for a divorce and refused to move in with her husband. Her husband abducted her and kept her in a locked house for a week, raping her several times. Before this incident, on a family trip, she was forced to spend a night with her husband and this was the first time ever she was alone with a man:

I was scared, so scared… He brought a pillow and asked me to come to bed. I said no. He dragged me and threw me on the floor and molested me until he jerked off, enjoyed it and, without cleaning his mess, slept. I was shouting! “Leave me alone! Don’t do that!” But he didn’t listen. It wasn’t sexual intercourse, but it the first time I saw such a scene. I would throw up when I remembered the feeling of touching his body.

Sara was forced to marry when she was 16 because of family poverty. She described herself as a sex slave in that marital relationship because of the continual forced sexual intercourse. Sonia, Tara, and Tania were in love with their partners before marriage; however, love could not prevent violence in their cases. Sonia had known her ex-husband since childhood, as they were neighbors. She fell in love with him when she was 14 and they got married when she was 16, without her parents’ agreement. They moved in together 2 years later. Her ex-husband did not respond to her sexual needs and frequently told her that he wanted her only for sex, so they would have sex only when he was ready: “He didn’t care about me… I felt I was a slave.”

Tara fell in love with a much younger man on a family trip. She was 8 years older than her husband, which was controversial in both families, and Tara’s family only accepted their marriage because she insisted on marrying him. Tara knew that her husband was addicted to methamphetamine:

He didn’t care about what I wanted. He would jerk off then roll over to another side of the bed, turn his back to me and say “You’re too ugly. I feel sick when I look at you. I just need you to jerk off.”

Tania was also in love with her husband before his violent acts began. She had applied for divorce after just 4 months after marriage because of his constant physical abuse and forced sex.

#### Sub-theme: Unusual/Uncomfortable Sexual Activities and Requests

Unusual sexual acts or requests were other types of sexual violence the women disclosed. These incidents happened in all cases except for Mina, who filed for divorce before moving in with her husband. The majority of participants complained about their husbands watching porn and then requesting similar positions or acts during sex. Sara was religious and watching porn was not something she could tolerate. However, her ex-husband would watch porn and then force her to imitate porn stars:

When we had sex, I felt I was sleeping with an animal. He would tell me what he had seen in the porn clips and force me to imitate the women. I was annoyed by his unpleasant and sinful acts, but I would try to cooperate… One time I fainted due to pain...

Tina and Sima mentioned their husbands’ desires to get involved in group sex. Tina said: “he wanted violent as well as group sex. I always refused and he would beat me up for that. He would call his friends during sex and tell them what he was doing.”

Sima had infertility issue and after some years she agreed that her husband could remarry. After her husband got married, he asked Sima to have threesome sex, which Sima did not agree to. However, she became involved in this kind of act after she was beaten up and forced to be in the relationship.

In Islam, sex during the menstrual period is prohibited. However, Sonia’s ex-husband did not care about this and forced her to have sex during her period:

He would do it [sex] when he wanted it. Even if I was having my period I had to listen to him and satisfy him… When I refused he would say I only want you for sex, only sex has any meaning for me.

Humiliation was another experience almost all participants mentioned. Tara was 8 years older than her ex-husband – she was 28 and he was 20 – and shortly after they got married he started to bring their age difference into a conversation, saying that “You are too ugly and old. I can’t have good sex with you.” Tara could not tolerate his sexual activities, which included phone sex with other girls in front of her while putting the phone on speaker and forcing her to listen to the conversation. After finishing the phone call, he would talk about the women’s bodies and what they would do for him and request Tara to do the same. When Tara objected to this behavior he would say “I’m ashamed of going out with you because you are ugly and old.” Calling Tara by other women’s names during sex was another degrading and humiliating experience that Tara mentioned: “When we had sex, he would call me by other names.” The most humiliating incident for Tara was when her now ex-husband brought a girl home:

One day he came home with a woman. He told her that I was his maid, then whispered in the woman’s ear “She is my wife” and then they both laughed… They went to the bedroom and he told me if I said a word then he’d kill me later… I was so disturbed hearing their laughter… After a while, he shouted my name and asked for me… I was forced to go into the bedroom and see them in a disgusting position and to make a drink for them. I’d like to have killed him, but I was so scared...

#### Sub-theme: Risks and Danger

Threats were a part of all incidences of sexual violence. A range of threats, from harming the woman to harming their relatives, was disclosed in the women’s narratives. Mina received a warning from her husband when she did not agree to move in with him. He threatened to abduct and rape her, which he eventually did, as well as threatening to kill her or to attack her with acid if she did not agree to move in with him. She was also scared for her family as her husband frequently threatened to harm or kill them if they did not make her move in with him. “I’ll kill you, and I won’t let you be someone else’s. I’ll burn your face with acid,” she remembered him saying. Mina filed for divorced before moving in with him in the end.

Sara narrated one of the most disturbing threats of all of our participants’ life stories. As mentioned earlier in this paper, her husband threatened her with a knife to make her do similar things to what he had seen in porn movies and also threatened to rape their daughters if Sara did not accede to his wishes. Tara remembered several incidences where her husband threatened her with death. She was also forced to have an abortion and threatened with a knife if she did not terminate the pregnancy.

Sonia lost her child after being physically attacked by her husband:

He did not want the baby and several times he made an appointment for me to have an abortion. He was forcing me to terminate the pregnancy, but I wouldn’t listen. Then one day he hit me in my belly several times until I fell down on the floor, bleeding. I lost my baby.

Tina and Tania’s husbands took nude photos of them and threatened that they would distribute the photos if they did not comply with their requests to have group sex. Some participants were scared of losing custody of their children, as child custody in Iran is the father’s rights.

All of the participants were in danger of and reported experiencing marital rape; however, only three of them actually used the Persian word for rape, which is *tajavoz*. According to Mina: “Tajavoz, tajavoz, tajavoz [rape] was terrible for me.”

Sara remarked: “I was like a sexual slave, just a resource for my husband’s sexual desires. I would cry a lot and I felt he was raping me.” Tara left her husband when he tried to suffocate her. “Every night I had to feel him on top of me, raping me,” she said.

Sonia did not use the word *tajavoz*; however, she mentioned the word *bardeh jensi* [sexual slave] to refer to her experiences. Other participants used words that depicted violent sexual acts as non-human behaviors; *like an animal* or *like a dog* were some descriptors the majority of participants used to refer to their husbands’ sexual activities.

#### Sub-theme: Effects of Sexual Violence

Participants talked about a variety of resulting emotional distress and physical harm related to the sexual violence that they had experienced.

All of the women experienced physical and emotional abuse along with sexual violence that needed medical care at some point in their marriage. Sara’s young body could not tolerate the uncomfortable sexual positions and felt pain in her body much of the time she: “It was too painful, and one time I fainted from the pain [during sex].”

All participants felt that they had been humiliated and degraded by their husbands, in different ways, and they referred to the emotional problems they experienced during their marriage. All felt low levels of self-esteem and self-confidence and low levels of control in their lives. They talked about feelings of helplessness, hopelessness, and being alone; feeling depressed, and described trauma-related problems and symptoms of post-traumatic stress disorder (PTSD).

Distrust of other people, even their relatives and parents, self-blame, feelings of having no security at home and the perception that they were living with an animal were some other emotional issues the women reported.

Sonia said:

I loved him, but he didn’t care. I felt worthless and a had a deep feeling of failure… After the divorce, for a long time, I couldn’t sleep without a sleeping pill and I also started smoking. I broke ties with my friends. I didn’t trust others because I felt everybody wanted to abuse me.

Tara constantly suffered different kinds of abuse, right from the very start of her marriage. Although they were in love, as mentioned earlier, shortly after their marriage her husband started to humiliate her, saying she was ugly and old:

He was very beautiful, and I really loved him. Hearing these words from him was too painful for me…now I hate men, hate sex, hate myself, hate life… Sometimes I’d feel I was nothing, I had no value, and I hated being a woman.

Suicidal ideation was another emotional problem the women experienced because of the constant violence they endured. Five participants mentioned that they had had suicidal thoughts when they were living with their husbands, and two of them had a history of attempting suicide.

He would rape me then go to sleep somewhere and snore like a bear. I felt sick and wanted to kill myself…I lived in fear for four years…and even now I still live in fear and have nightmares. Sometimes I hate being a woman. I think I was just an object to be used (Mina).

### Theme 2: Empowerment, Hopes, and Hurdles

Although the participants faced serious sexual violence in their marriages and felt helpless and hopeless at certain stages in their lives, they were not passive. Eventually, they all tried to get help from their social networks or from formal agencies. At first, nobody took their requests for help seriously and they often had to be the victim of several violent incidents before other people would believe them.

#### Sub-theme: Speaking out Against the Perpetrator

Fear was the first emotion all women mentioned as their reason for not speaking out against their husbands’ violent actions. As explained above, the men would threaten their women in different ways and the women experienced grave fear for their lives, their loved ones’ lives, and losing their children, as well as divorce and losing their status in society.

Although our participants felt helpless and hopeless and were scared for their lives, eventually they objected to the ways their husbands behaved.

Mina frequently told her husband that she did not like him and that she wanted a divorce:

I told him that I didn’t like him, but he said he loved me and that that was enough. I told him “I am not scared of you. You can hit me, shout at me, or kill me; but I don’t feel scared anymore.”

Mina’s brave behavior happened in the context of physical abuse and knowing that her husband was capable of what he had threatened he would do to her. Despite this, she overcame her fears and spoke out against him.

After 7 years of tolerating sexual violence, Sara decided to refuse to have sex with her husband. However, she was forced to resume sexual activities because of her husband’s threat to rape their daughter, after which she finally filed for divorce.

Sonia was verbal and asked for more intimacy and foreplay during sex. She would say “no” when she did not agree with something, although her husband would continue with his behavior, regardless, each time. Tara was the only participant who did not speak out against her husband. On two occasions her husband followed her with a knife and tried to kill her, so Tara was very scared of speaking out against him. She would run away or leave home when she felt she was in danger.

Kiana was scared of divorce and not having enough support after the divorce. Her husband and his family often threatened her with divorce telling her that, *if you’re not happy, pack up your things and go away*.

#### Sub-theme: Help-Seeking Behavior

As mentioned earlier, women were not passive when they were exposed to sexual violence. Social networks were the first resource they employed to save themselves from the violence, and family members were the first point of contact for all of the participants. All of the women except Kiana sought help from official authorities after several attempts at making their parents or relatives agree they should do so. In the case of Kiana, her father had passed away and her mother struggled with depression, so she felt she did not have enough family support to go through separation or divorce. Instead, she started counseling to make herself empowered enough to tolerate her life.

Parents, siblings, close relatives, neighbors, and friends were the main resources that participants turned to when seeking help. At the start, none of them received the support they needed and expected, leading them to feel disempowered, helpless and hopeless. Parents and other people often did not hear the women’s cries for help. “Go and live with him, you will love him,” “have sex with him, then you will love him,” “I didn’t like your dad, but after some time I became more interested in him,” “all men are like him,” “this is how it should be,” “he is a man,” “feed him and have sex with him and he will get better,” “be silent,” and “don’t tell anyone” were some recommendations the majority of participants received at first. Soha’s neighbor told her to shut up and live with her husband because they believed “all men were like him.” The neighbors suggested her to bear some children so he would be better.

Social networks had a strong role in accepting or refusing the cries for help and in all cases in this research except one, everybody agreed that divorce might save the women after several months or years of tolerating violence.

After marriage and facing violence Sara said:

I left home several times, but my mom sent me back saying I don’t have enough money for you or saying that he is your husband, it [sexual pleasure] is his right… all men are like him.

However, in the end her parents were the ones that asked Sara to get a divorce, when they found out that she was working in other people’s houses as a maid and believed that she was living under the threat of severe violence.

Sonia and Tara felt they could not ask for help because they got married willingly and at their own insistence. Sonia threatened to kill herself if her parents did not agree to her marriage and Tara’s parents only accepted their marriage because of her persistence. Both said that they could not seek help because everybody would say that it was their choice. Sonia said:

I was so sad because my parents were not supportive at all. My mum would say this is what you wanted, he is a man and you have to cope with him. It’s bad for our reputation if you get a divorce.

Tara took refuge at her parent’s house for several months until her husband came to pick her up. She went back with her abusive husband because her parents did not agree with divorce. “What would other people say behind our backs,” was a common reaction that most participants heard from others.

Tala and Tanina received the most immediate help, gaining support from their parents from the start. Tanina said: “my uncle told me he [her husband] doesn’t have any right to beat you up,” and Tala said that: “my family always supported me.”

All of the women talked about a sense of empowerment or disempowerment in their narratives. As mentioned earlier, when they did not receive social support, the women felt helplessness and hopelessness; however, all of them were finally able to make their parents and relatives see that their marital relationships were full of violence and that they did not feel safe in their homes. Participants felt strong and brave when they found the courage to speak out against their violent partners, or when their parents supported their desire to return to their parents’ houses.

All of the women sought help from formal services at some point. As mentioned, participants were recruited from counseling centers and a healthcare center; however, all went to these centers after several years of tolerating violence, leading to this conclusion that counselors and legal authorities were the last resort for the women in this study.

## Discussion

Participants in this research were exposed to several kinds of sexual violence including forced intercourse, unusual and/or uncomfortable sexual activities and requests, and being made to have feelings of risk and danger in their marital relationship. In line with national and global research findings (Fallah et al., 2015; Pengpid et al., 2018), participants disclosed a mixture of physical and emotional abuse while they were experiencing IPSV, leading to physical problems including pain and even abortion, and emotional problems such as feelings of disempowerment, dehumanization, being degraded, depression, and a sense of being worthless.

How women disclose IPSV and seek help depends on an interaction between cultural scripts and the “victim’s” level of support or perceived support from their family. Support from parents, friends, and relatives creates feelings of empowerment, while a lack of social support creates emotional hurdles. Family and friends can rule what a woman discloses and close the door to seeking further help, or they present an informal help-seeking network, taking a more helpful role and offering shelter and support. Sometimes it can take a long time for a family to believe how serious a situation is and that can lead to emotional problems for the victim.

All participants talked about their fear of violence at some point of their lives and how they had tolerated it. When they started to seek help they all sought help from their parents and sisters as their first port of call. This is in line with Kleinman’s (1980) explanation of help-seeking behaviors around the world. He argued that the majority of help-seeking behaviors in both Western and non-Western countries make use of popular sectors or social networks. Therefore, when people encounter a problem, family and social networks are the first domain wherein the problem is revealed, perceived, and labeled. Family and social networks are the first who get to decide what should be done about the problem and how to treat it. More recent data corroborate Kleinman’s argument that at the time of health-related issues, family and social networks are the most important resources for help (Dejman et al., 2010; Donnelly et al., 2011).

Seeking help did not necessarily solve the problem, as participants described. Cultural rules and customs play an important role in defining, justifying, and dealing with IPSV. In line with feminist approaches, the patriarchal structure of Iranian culture, especially among the older generation, endorses the idea that a woman should be submissive and ready to satisfy her husband’s sexual desires under any circumstances and at any cost. This idea led to our participants’ cries for help as being perceived as something not very serious. Almost all participates were given similar justifications for their husbands’ actions: he is a man, a man should be like him, all men are like him, or men have greater sexual desires. Social justification of sexual violence as a normal act that is related to men’s higher sexual needs often prevents women from seeking help or makes them underestimate the seriousness of the situation. This finding has been replicated in recent studies in other countries with traditional patriarchal views (Adinkrah, 2017; Lynch et al., 2017; Odwe et al., 2018).

All participants except one tried to get divorce at some point in their marriage and asked their parents to support them. Their parents’ objections to divorce rise from the cultural scripts around keeping everything in the family, placing the family’s reputation first, and women’s responsibility to preventing marriage break-down for the sake of her family’s reputation, and for the sake of the children (where appropriate). One surprising issue was that sending women back to live with their husband happened in both the arranged and the love-marriage examples. In arranged marriages, families were the supporter of the marriage in the first place and thus insisted on keeping the marriage alive after problems began, although in the end most of the parents then supported a divorce as well. In the love-marriage cases, seeking help resulted in the same answer: “It was your choice.” There are several Iranian expressions with a meaning that one should take responsibility for his/her action. Hence, in the cases of love marriages, the participants’ parents felt no responsibility to interfere and stop the violence, so the participants felt there were no resources for them to seek help. In short, from the participants’ points of view, a lack of support from the family was one of the most important obstacles to seeking help, and instead family and friends would justify violence as being something normal.

In line with previous research findings (Issahaku, 2015; Hahm et al., 2017; Honda et al., 2018; Pengpid et al., 2018), these women who had experienced abusive relationships reported physical pain, a low level of self-esteem and self-worth, depression and trauma-related symptoms. A sense of disempowerment occurred in particular when they felt that there was nobody to help them and they had nowhere to go. Although IPSV took its toll on the emotional wellbeing of the participants, all of them felt empowerment and had a sense of self-efficacy when they received social support. They felt they were now more mature and stronger and can achieve many things because they have survived their horrific experiences. This is a promising finding for both survivors of IPSV and the professionals working with them. As participants mentioned, the timing is an important factor. Many women in abusive relationships stay silent for a long time and this would lead to physical and psychological concerns for them. Psychologists, counselors, and social workers can close this time gap by creating programs to help women in abusive relationships to build the necessary support network or to use existing network in seeking help. Creating preventive programs that talk about the possibility of sexual violence in a marital relationship is recommended as in some families and societies, marital rape has not been recognized yet.

## Data Availability Statement

The datasets generated for this study are available on request to the corresponding author.

## Ethics Statement

This study was carried out in accordance with the recommendations of the University of Isfahan Ethics Committee for the Department of Counseling. All subjects gave written, informed consent in accordance with the Declaration of Helsinki. It contained a brief description of the research and informed the participants that the interview would be audio-recorded; four participants refused to be audio-recorded and the interviewers took notes during the interview instead. In this study, pseudonyms have been used to ensure confidentiality. The interviews were conducted at a time and location of the participants’ choice.

## Author Contributions

AN developed the idea, supervised the qualitative interviews, performed two qualitative analyses, and wrote the manuscript. SA and MB performed some of the qualitative interviews and assisted in the qualitative analysis. JD supervised data analysis and writing of the manuscript. All authors discussed the results and contributed to the final manuscript.

### Conflict of Interest

The authors declare that the research was conducted in the absence of any commercial or financial relationships that could be construed as a potential conflict of interest.
